# Randomized trial of a novel game-based appointment system for a university hospital venereology unit: study protocol

**DOI:** 10.1186/s12911-015-0143-9

**Published:** 2015-04-08

**Authors:** Elia Gabarron, J Artur Serrano, Luis Fernandez-Luque, Rolf Wynn, Thomas Schopf

**Affiliations:** NST-Norwegian Centre for Integrated Care and Telemedicine, University Hospital of North Norway, N-9038 Tromsø, Norway; Department of Clinical Medicine, Faculty of Health Sciences, UiT - The Arctic University of Norway, Tromsø, Norway; NORUT – Northern Research Institute, Tromsø, Norway; Salumedia.com, Sevilla, Spain; Division of Mental Health and Addictions, University Hospital of North Norway, Tromsø, Norway

**Keywords:** Sexually transmitted diseases, Chlamydia, Health promotion, Health education, Public health informatics, Health information technologies, Internet, Social network, Social media, Young adult, Adolescent

## Abstract

**Background:**

Chlamydia is the most common reportable sexually transmitted disease (STD) in Norway, and its incidence in the two northernmost counties has been disclosed to be nearly the double of the Norwegian average. The latest publicly available rates showed that 85.6% of the new cases were diagnosed in people under 29 years old.

The information and communication technologies are among the most powerful influences in the lives of young people. The Internet can potentially represent a way to educate on sexual health and encourage young people, and especially youth, to be tested for STDs. If hospital websites include an easy and anonymous system for scheduling appointments with the clinic, it is possible that this could lead to an increase in the number of people tested for STDs.

**Methods:**

The purpose of the study is to assess the impact of a game-based appointment system on the frequency of consultations at a venereology unit and on the use of an educational web app. An A/B testing methodology is used. Users from the city of Tromsø, in North Norway, will be randomized to one of the two versions of the game-style web app on sexual health at www.sjekkdeg.no. Group A will have access to educational content only, while group B will have, in addition, access to a game-based appointment system with automatic prioritization. After one year of the trial, it will be analyzed if the game-based appointment system increases the number of consultations at the venereology unit and if health professionals deem the system useful.

**Discussion:**

This study will explore if facilitating the access to health services for youth through the use of a game-based appointment system integrated in a game-style web app on sexual health education can have an impact on appointment rates.

**Trial registration:**

The trial is registered at clinicaltrials.org under the identifier ClinicalTrials.gov NCT:02128620

## Background

Chlamydia is the most common reportable sexually transmitted disease (STD) in Norway. The two northernmost counties had the highest chlamydia rates in Norway in 2012 [1]; as exemplified by the numbers in Finnmark county, where the incidence was nearly the double of the Norwegian average. In 2012, 21.489 people were diagnosed in Norway with genital chlamydia [1], 85.6% of them were under 29 years old [1], and analyses of the chlamydia testing rates have shown that the frequency of testing is lower in the youngest age group, which has the highest risk of infection [2].

It is relevant to facilitate the chlamydia testing for youth (15-24 years old) since up to 80% of the patients infected have few or no symptoms, and for some of those the long-term consequences of untreated chlamydia infection are infertility and chronic pelvic pain. Many young people lack knowledge about STDs. They are more aware of the risks of unwanted pregnancy than their risk of acquiring a STD [3]. Youth has a great need for advice and guidance on contraception and STDs. While most families think that youth should receive sex information from their parents, the main source of information for youth actually comes from the media [4]. Online information and social networks may therefore be helpful in making youth more aware of STDs [3]. These media are among the most powerful influences in the lives of young people [5-7], and a Norwegian survey performed in the 2^nd^ quarter of 2011 showed that 100% of youth from 16 to 24 years old use the Internet every day or almost every day, mainly for social networking purposes [8].

The use of information and communication technologies related to sexually transmitted diseases has been studied during recent years [9-13]. We now know that the Internet can be used to promote safer sexual practices [14-16]. A bibliographic review [16] showed the efficacy of computer-based, Internet-based and mobile phone-based interventions for STD prevention and treatment support. New technologies can transform targeted, routine, and consumer-controlled STDs testing as well as partner interventions [16]. Potentially, a higher number of young people, and especially youth, would be tested for STDs if those online applications included an easy to use and anonymous system for obtaining appointments with a clinic.

Young people may feel insecure when they have to talk to health professionals on matters concerning their sexual health [17]. It is a great challenge for youth to book an appointment with a health professional, which often involves a phone conversation with a nurse or medical secretary who asks the reason for the appointment [17]. A prior trial [18], which tested online appointments for patients with suspected sexually transmitted infections, showed a high degree of user satisfaction [18]. However, the system notified the patients about the date and time of the appointment after several hours, up to one working day [18]. This may limit the number of requests for an appointment. A system that provides the user the date and time of the appointment immediately might further increase the demand for appointments.

The main objective is to assess if a game-based appointment system, with an automatic prioritization algorithm, integrated in the already existing game-style web app on sexual health education www.sjekkdeg.no including social media [10] can facilitate the access to health services for youth; as well as to analyse its perceived usefulness by health professionals.

## Methods/Design

### Trial design

To assess the impact of the game-based appointment system, an A/B testing methodology is used. Two versions of the web app, www.sjekkdeg.no, will be used for the A/B test to explore the user interactivity with the site. Version A (control), consisting of the educative web app only; and Version B, which will additionally include a game-based appointment system. Users will be randomly selected to use one of the two versions of the web app. Thus, we will be able to assess which version better supports the typical user in tasks such as booking an appointment at the hospital venereology unit.

The novel game-based appointment system is integrated in the already existing web app www.sjekkdeg.no [10], a game-style web app on sexual health education including social media, targeting North-Norwegian youth, accessible through laptops, smartphones and tablet computers [10]. Gamification techniques [19] such as avatars, achievement-based gifts and social network sharing buttons have been implemented in www.sjekkdeg.no. The web app, which was launched at the end of 2012, seems to be a promising way to encourage users to learn more about sexual health, judging from preliminary visitor returning rates, the average pages visited and the time spent on it [19]. The data also show that the symptom checker is one of the most visited components of the web app [19].

The new game-based appointment system, made available to the users that have been randomized to the intervention group, is based on an automatic prioritization algorithm, which provides the opportunity to book an appointment online. Once randomized to the intervention group, a virtual doctor will ask questions concerning the symptoms that the avatar has experienced. According to the avatar’s answers, the virtual doctor suggests a possible diagnosis or, if a diagnosis is not possible, that the avatar should attend the clinic to be tested for STDs. The appointment system service will be available 24/7, from 1^st^ January 2015 until 31^st^ December 2015.

The system was reviewed by a panel of specialists in venereal diseases. They contributed in the definition of the relevant symptoms that influence the priority attributed to the patients by the system. We envisaged three levels of prioritization (i.e. triage): 1) Emergency appointments: The user should see a doctor on the same day or the following day. Patients having fever or other signs of serious infection are assigned to this category. They are strongly advised to call the clinic by phone and should not use the game-based appointment system. 2) Haste-appointments: The user should see a doctor within 3-4 days. Typical cases are acute infections with itch or pain. 3) Routine-appointments: The user should see a doctor within 2-3 weeks. Most cases in this category are asymptomatic infections.

**Figure 1:**
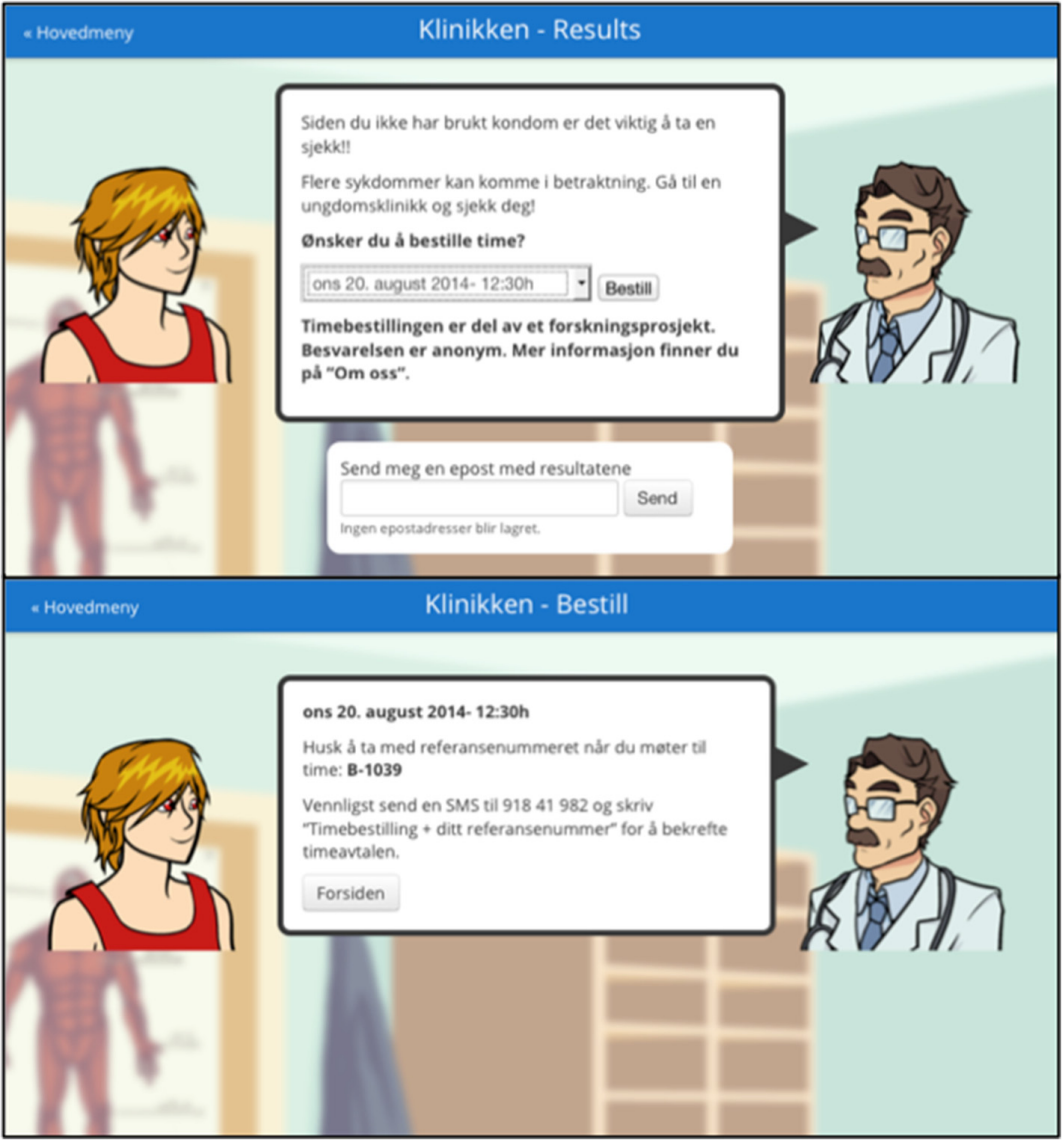
**Example of the user’s screen to book an appointment.** Screen captures from www.sjekkdeg.no.

**Figure 2:**
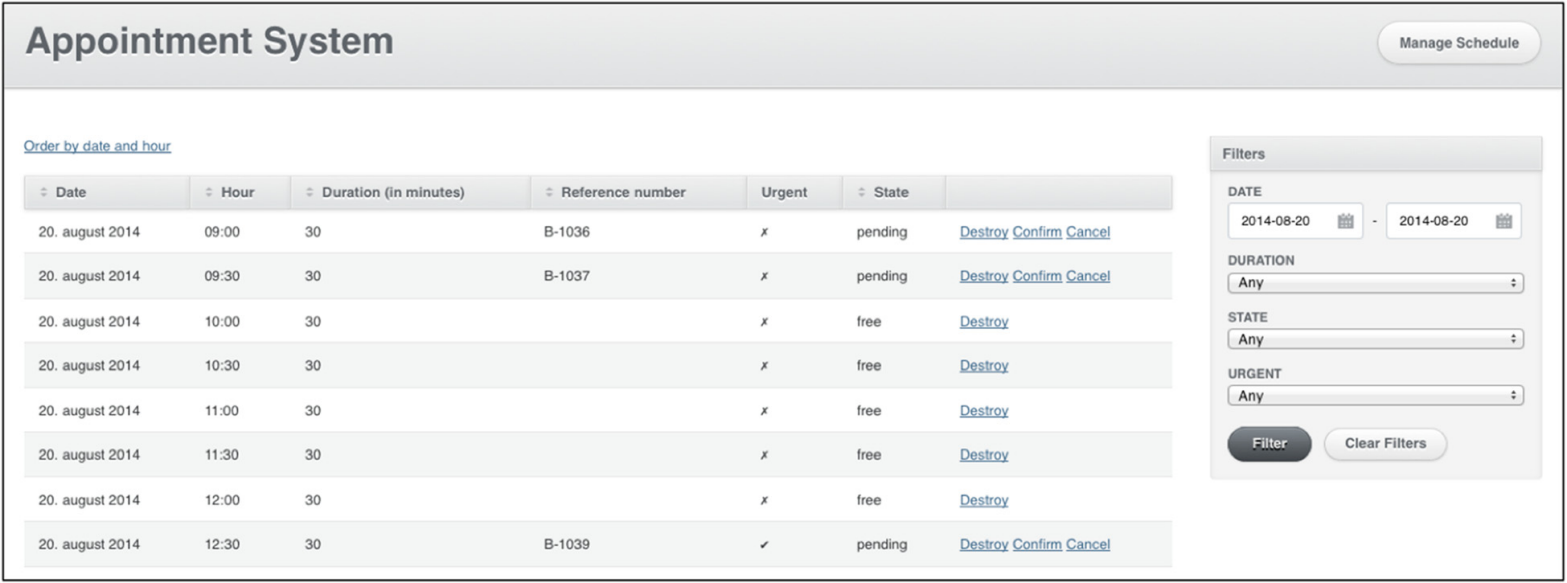
**Health professionals’ administration dashboard.** Screen capture from www.sjekkdeg.no administration dashboard.

To book an online appointment the user will require a reference number that will appear jointly with the guided diagnosis provided in the symptom checker. Example of the user’s screen to book an appointment can be seen in Figure 1. This reference number will be the user’s “credentials” at the clinic. Simultaneously, the venereology unit will get an automatic notification with the provisional pre-selected day and time for the appointment, and the user’s symptoms report, which could be relevant for planning the workflow at the clinic. The appointment at the venereology unit is accepted by the health professionals once the user sends a SMS confirming his/her intention of showing up. Figure 2 shows the health professionals’ administration dashboard.

The appointment system is designed to preserve the user’s anonymity. Thus, users will not need to provide their name or other personal data to use the appointment system. Only the health professionals at the venereology unit, at the University Hospital of North Norway, will be able to identify patients before their appointments through their cell phone numbers.

The users who ask an appointment through the web app, and who come to the visit at the venereology unit will be asked to sign an informed consent.

To our knowledge, a sexual health web-based app with this type of functionality has not been reported in the literature.

### Participants

The web app www.sjekkdeg.no is of free use, and accessible to everyone, although only the users from the city of Tromsø are eligible to participate in this study.

**Figure 3:**
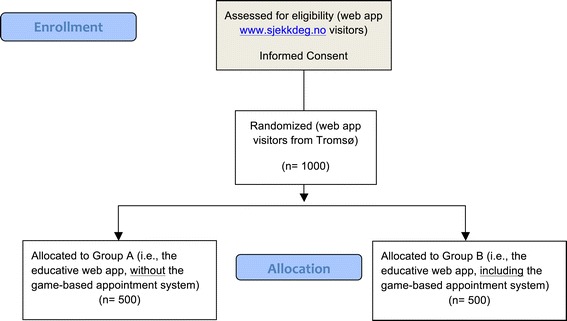
**Study flowchart.**

Once a user accesses the web app www.sjekkdeg.no, an informed consent appears on the screen. If the user agrees to participate in the research project, then, a question asking his/her location will appear, as follows: “All the content on this web app is completely anonymous. For research purposes, we only would like to know if you are from Tromsø.” There are two possible answer-options: “Yes, I am from Tromsø”; or “No, I am not from Tromsø”. Users selecting the option “No, I am not from Tromsø” will be immediately selected for the Group A or control group (i.e., the educative web app, without the game-based appointment system). Users answering, “Yes, I am from Tromsø” will be randomized either to the Group A, or to Group B (educative web app, including the game-based appointment system), with a 1:1 allocation ratio (See the study flowchart in Figure 3).

All web app users, either belonging to Group A or Group B, that use the symptom checker functionality will receive individual advice, e.g. on infection prevention measures such as advice on how to use condoms until the applicant has tested him/herself for STDs or otherwise clarified his/her condition. The individual advice will be based on the information that the users have provided regarding their symptoms.

### Intervention

The intervention group will be able to use a game-based appointment system in the web app, allowing them to book an appointment at the hospital venereology unit, in addition to all the educational content of sjekkdeg.no.

The control group will have access to the educative web app only.

### Outcomes

#### Primary outcome

The primary outcome is the number of consultations with health professionals. We expect the number of visits with health professionals will be higher in the game-based appointment group (Group B) when compared to the control group (Group A).

To assess if the game-based appointment system increases the number of consultations on STDs at the venereology unit at the University Hospital of North Norway, we will analyse the total number of appointments booked through the web app within one year after implementation and compare this data with earlier appointments rates. We will compare the total number of STDs consultations belonging to Group A versus the total number of consultations attended through the game-based appointment-system (Group B) within one year of implementation (quantitative approach). To compare the total number of STDs consultations, users in the control group are asked to bring their symptom checker results including their randomization code, to the visit at the venereology unit. Attended visits at the venereology unit of users showing randomization codes belonging to the control group will be registered in a grid.

#### Secondary outcomes

Secondary outcomes are: 1) the impact of the game-based appointment system on how the users utilise the web app www.sjekkdeg.no; 2) the potential benefit for health professionals (doctors and nurses) from such a system through reduced consultation times due to previously acquired information by the symptom checker; and 3) the potential benefit for the users from this game-based appointment system by reducing the booking time threshold of a consultation at the venereology department at the hospital.

On the impact of the game-based appointment system on how the users utilise the web app www.sjekkdeg.no, we expect that the number of visits to the educative components of sjekkdeg.no will increase, after the implementation of the game-based appointment system functionality. And also, we expect that the number of visits to the educative components of sjekkdeg.no will be higher in the appointment group when compared to the control group.

To assess if the game-based appointment system increases the number of visits to the educative components of the web app www.sjekkdeg.no, we will analyse and compare the absolute number and percentage of visits (new visitors and returning visitors) of users in Group A versus Group B after one year of the start of the project. The total number of visitors, as well as the number of new and returning visitors, included in both randomization groups, using the web app, and all of its components, will be tracked through the web analytics tool Google Analytics.

Regarding the potential benefit for health professionals (doctors and nurses) from such a system; we expect that health professionals will be satisfied by having reduced consultation times due to previously acquired information by the symptom checker. To assess if health professionals (doctors and nurses) can benefit from such a system (e.g. through reduced consultation times), a qualitative approach consisting of an *ad hoc* questionnaire based on the Technology Acceptance Model [20], will be used.

On the potential benefit for the web app users; we expect that the time spent per visit to the web app will be longer in the appointment group than in the control group, implying a higher exposure to health information. And we also expect that the visitors’ returning rate will be higher in the appointment group when compared to the control group. To assess whether the game-based appointment system influences how the users utilise the web app www.sjekkdeg.no, we will compare, after one year of implementation, how much time the users spent on the web app, and on each educative component. To assess if the users can benefit from the game-based appointment system and how this functionality helped them in the decision to book an appointment with the doctor, a qualitative approach, consisting of an *adhoc* questionnaire based on the Technology Acceptance Model [20], will be used. The questionnaire will be available at the venereology unit, before the consultation.

### Sample size

The web app www.sjekkdeg.no has been running for more than one year and has had, so far, more than 800 unique visitors from Tromsø. We expect the use of the web app to increase further, as it gradually becomes more known. This allows us to assume that one year will be enough to conduct the study and have 1000 visitors.

If 1000 www.sjekkdeg.no users are randomized into the two groups, Group A and Group B, and approximately half of the users in Group A attend an appointment, we can detect a 20% change in the appointment rate (with beta = 0,80 and alpha = 0,05). If the number of appointments attended by users in Group A is lower than expected, the difference between the groups must be larger for us to detect it. With a 30% attended appointments rate in Group A, we still have power to detect a 30% difference between the groups (with beta = 0,80 and alpha = 0,05).

### Interim analysis and stopping guidelines

Because of the educational nature of the research study, no interim analysis or stopping guidelines are planned.

### Randomisation

The users from Tromsø will be randomized either to the Group A, or to Group B, with a 1:1 allocation ratio (See the study flowchart in Figure 3). Simple randomization will be used. The random allocation sequence will be generated automatically using Google Analytics randomizer code.

Web app users and health professionals are not blinded.

### Statistical methods

Means, standard deviations and frequencies will be used to describe the number of attended visits at the venereology unit with a randomization code (sjekkdeg.no web app users), as well as the absolute number and percentage of visits (new visitors and returning visitors), and the time spent in every educative component among web app users in both groups, after one year of the start of the project.

T-tests will be used to compare the number of appointments attended by users in both groups; and also to compare the number of sjekkdeg.no visitors (new visitors and returning visitors), and the time spent in every educative component among users in Group A versus Group B.

Data will be analysed using the Statistical Package for the Social Sciences (SPSS version 22).

### Ethics

The research activities will strictly follow the regulations provided by the Health Register Act (Helseregisterloven) [21] and the Health Personnel Act (Helsepersonelloven) [22]. Both acts (with accompanying regulations) being supervised by the Norwegian Ministry of Health and the Norwegian Board of Health. To guarantee approval regarding any possible use of data in a personal way, this project will pursue conformance to the regulations of the Personal Data Act [23] (based on the European Data Protection Directive [24]). The Personal Data act and other relevant acts and regulations are enforced and supervised by the Norwegian Data Inspectorate (Datatilsynet). The study has been approved by the Privacy Ombudsmann at the University Hospital of North Norway.

The project protocol was presented for assessment to the Norwegian Regional Ethics Committee (REK-Nord), with the reference code: 406678, and it was declared exempt.

## Discussion

This study will explore if facilitating the access to health services for youth through the use of a game-based appointment system integrated in a game-style web app on sexual health education, can have a positive impact on consultation rates.

The premise guiding the development of the game-based appointment system is that information and communication technologies may facilitate the access to health services for youth regarding sexual health and the prevention of STDs, as well as increase their knowledge about STDs. And the early triage of patients may be an effective way to reduce health care staff resources and expenses.

The project will provide a health related social network aimed at empowering citizens to better understand and become more aware of STDs, to prevent STDs, and to receive appropriate treatment for STDs [3].

### Limitations and generalizability

Because the trial is only carried out in the city of Tromsø, the results cannot be generalized to the Norwegian population. If the game-based appointment system proves to be a successful way to encourage young people to schedule appointments with the venereology unit at the University Hospital of North Norway, and to support the health professionals, then it could include additional venereology units in Norway, or even other health services targeting young people.

## Abbreviation

STD: Sexually transmitted disease
